# Nε-carboxymethyl-lysine promotes calcium deposition in VSMCs via intracellular oxidative stress-induced PDK4 activation and alters glucose metabolism

**DOI:** 10.18632/oncotarget.22835

**Published:** 2017-12-01

**Authors:** Wen-Qi Ma, Xi-Qiong Han, Ying Wang, Xin Wang, Yi Zhu, Nai-Feng Liu

**Affiliations:** ^1^ Department of Cardiology, Zhongda Hospital, School of Medicine, Southeast University, Nanjing 210009, P.R. China

**Keywords:** PDK4, CML, calcification, oxidative stress, glycolysis

## Abstract

Diabetes and vascular calcification are intrinsically linked. We previously reported that advanced glycation end products (AGEs) accelerate calcium deposition in vascular smooth muscle cells (VSMCs) via excessive oxidative stress. However, the underlying mechanism remains poorly understood. Pyruvate dehydrogenase kinase 4 (PDK4) is an important mitochondrial matrix enzyme in cellular energy metabolism. Since hyperactivation of PDK4 has been reported in calcified vessels and in patients with diabetes mellitus, inhibition of PDK4 expression may be a strategy for the prevention of diabetic vascular calcification. In this study, we used a rat VSMC model to investigate the role of PDK4 in diabetic vascular calcification and further explore the underlying mechanisms. We observed that Nε-carboxymethyl-lysine (CML), which is a major immunogen of AGEs, accelerated calcium deposition in VSMCs through PDK4 activation. An elevated level of reactive oxygen species (ROS) acted as a signal transduction intermediate to increase PDK4 expression. Either inhibition of PDK4 expression or RAGE (receptor for AGEs) blockade attenuated CML-induced VSMC calcification, as shown by decreased alkaline phosphatase (ALP) activity and runt-related transcription factor 2 (RUNX2) expression. Glucose consumption and lactate production were increased during CML-induced VSMC calcification. Importantly, CML accelerates glycolysis in VSMCs via a PDK4-dependent pathway. In conclusion, this study demonstrates a novel mechanism by which CML promotes VSMC calcification via PDK4 activation and alters glucose metabolism in VSMCs.

## INTRODUCTION

Vascular calcification, characterized by the deposition of calcium phosphate in cardiovascular tissue, is an active and cell-mediated process involving either the intima or media of blood vessels. It is associated with increased cardiovascular morbidity and mortality, especially in high-risk populations, such as patients with diabetes, atherosclerosis, or end-stage renal disease [1]. Although vascular calcification seems to be a uniform response to vascular impairment in different diseases, it is a heterogenous disorder with overlapping yet distinct mechanisms of initiation and progression [2]. The pathogenesis of vascular calcification is a complex multifactorial process, some of which includes elevated phosphate levels, inflammation, ageing, apoptosis, and oxidative stress [1, 3]. Vascular smooth muscle cells (VSMCs), the predominant cell type involved in medial vascular calcification, can undergo a phenotypic transition into osteoblast-like cells within the calcifying environment, as evidenced by increased expression of multiple bone-specific molecules, such as alkaline phosphatase (ALP), runt-related transcription factor 2 (RUNX2), and bone morphogenetic proteins [1, 4].

Diabetes and vascular calcification share several common pathogenic mechanisms and are intrinsically linked [5]. In diabetic patients, long-term hyperglycaemia is an important risk factor for severe cardiovascular complications, including accelerated atherosclerosis and medial calcification [6]. Clinical data have demonstrated the value of vascular calcification for the prediction of future cardiovascular events and death in patients with diabetes [7–9]. Furthermore, hyperglycaemia strongly promotes bone matrix protein expression and ALP activity [10–12].

One consequence of long-term hyperglycaemia is the formation and accumulation of advanced glycation end products (AGEs), especially in patients with serious diabetic complications [13]. Previous studies have shown that increased levels of AGEs augment the synthesis of reactive oxygen species (ROS) and result in excessive oxidative stress, which is believed to be responsible for diabetic vascular complications [5, 14, 15]. Excessive oxidative stress, in turn, further accelerates the formation of specific AGEs, including Nε-carboxymethyl-lysine (CML), which is a major immunogen of AGEs [16]. Particularly, diabetes-mediated vascular calcification exhibits several important factors, including oxidative stress and hyperglycaemia, that enhance AGE/RAGE (receptor for AGEs) signalling, which heavily influences both cellular and systemic responses [5]. In our laboratory, we have previously reported that AGEs increase oxidative stress during the calcification process [11]. However, the exact molecular mechanisms by which oxidative stress-mediated molecular signalling promotes vascular calcification has not been investigated in depth.

Pyruvate dehydrogenase kinases (PDKs) are mitochondrial regulators of cellular energy metabolism; PDKs inhibit pyruvate dehydrogenase (PDH) activity, which converts pyruvate either aerobically to acetyl-CoA or anaerobically to lactate [17]. Evidence has demonstrated the close link between PDKs and metabolic dysregulation under pathological conditions, such as insulin resistance and diabetes, obesity, cancer, and hyperglycaemia [18]. To date, four isoforms of PDK (PDK1-4) have been identified in humans, all of which exhibit tissue-specific expression. Among them, pyruvate dehydrogenase kinase 4 (PDK4) is highly expressed in vascular tissue, skeletal muscle, and the heart [19]. Recently, several publications have linked PDK4 to the process of vascular calcification. Lee *et al.* [20] reported that PDK4 knockout mice exhibit downregulated expression of osteoblast proteins and decreased calcium deposition in the aorta compared to wild-type mice and that PDK4 expression is increased in the calcified vessels of patients with atherosclerosis. In addition, increased protein expression of PDK4 is observed under diabetic conditions associated with dysregulated glucose metabolism [21]. However, nearly no data are available in the literature regarding the association between PDK4 and diabetic vascular calcification, although the abovementioned evidence effectively supports a role for PDK4 in inducing pro-osteoblastic effects and in glucose metabolism.

In this study, we investigated the role of PDK4 in CML-induced VSMC calcification and its possible mechanisms. Our results indicate that PDK4 is the downstream signalling molecule of CML-induced oxidative stress that stimulates calcium deposition in VSMCs by regulating the phenotypic switch of VSMCs to osteoblast-like cells. Furthermore, PDK4 participates in the switch of glucose metabolism during the calcification process.

## RESULTS

### Effects of CML on VSMC viability and apoptosis

**Figure 1:**
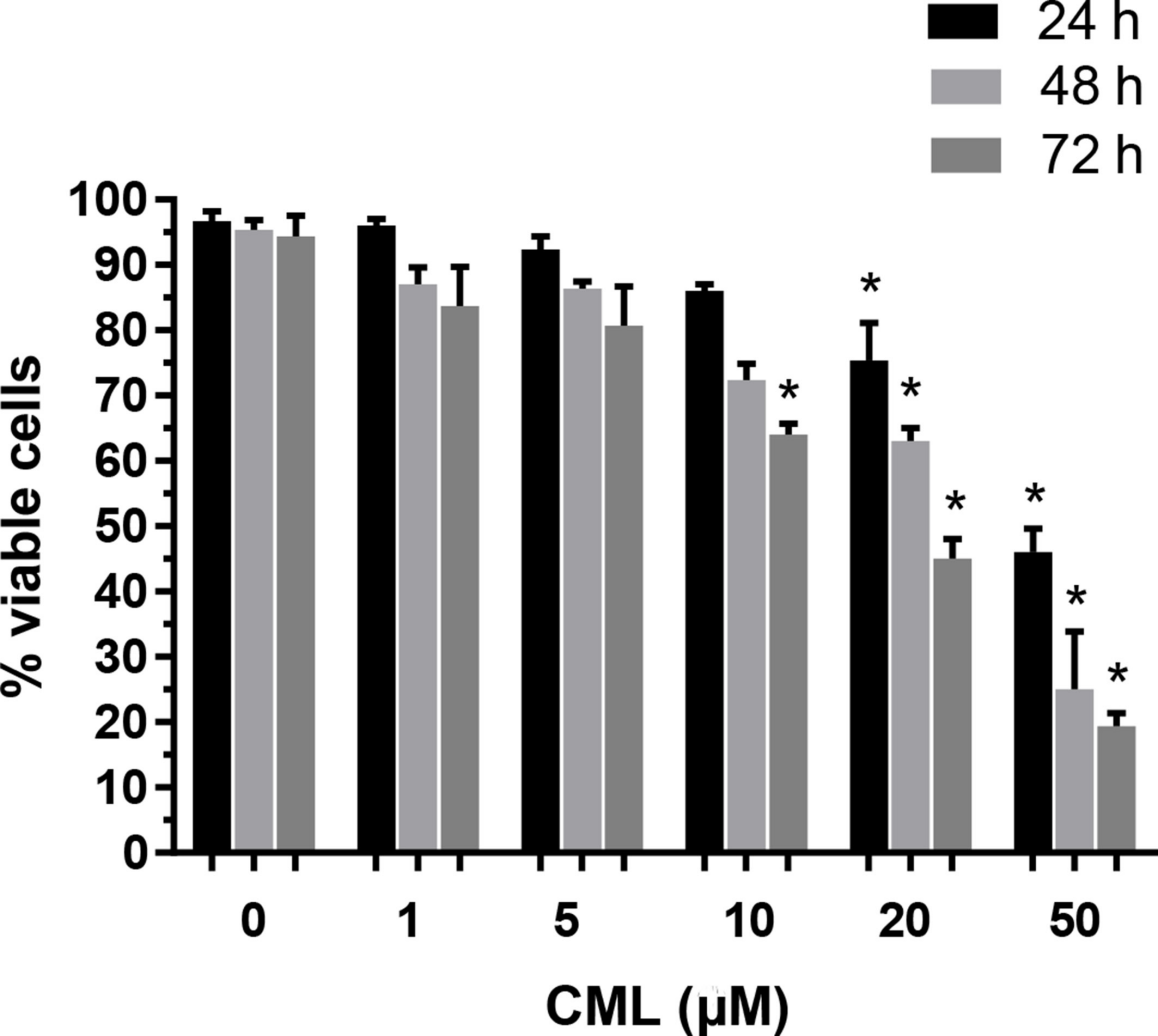
Effects of CML on VSMC viability VSMCs were incubated with increasing concentrations of CML (1–50 μM) for 24–72 h. The percentage of viable cells (quantitated by the MTT assay) was normalized against that in the control group. ^*^P < 0.05 compared to the corresponding control value.

VSMCs were treated with different concentrations of CML (1, 5, 10, 20, and 50 μM) for 24, 48, and 72 h. The MTT assay was performed to detect cell viability at different time points. As shown in Figure 1, treatment with 1–10 μM CML for 24 or 48 h did not significantly inhibit cell viability, whereas 50 μM CML inhibited VSMC viability in a time-dependent manner. Therefore, a dose of CML ranging from 1–10 μM and an incubation time of 24 or 48 h were selected for subsequent experiments.

Since apoptosis has been reported to be involved in the calcification process [22], we employed flow cytometry with annexin V and propidium iodide (PI) staining and the terminal deoxynucleotidyl transferase-mediated dUTP nick end labelling (TUNEL) assay to detect apoptotic cells and determine whether the effects of CML on VSMC calcification are caused by the activation of specific signalling pathways or by cell apoptosis. Annexin V/PI double staining showed that the VSMC apoptosis rate was not significantly increased after treatment with 10 μM CML for 24, 48, and 72 h (Supplementary Figure 1). Furthermore, the TUNEL assay also did not reveal excessive cell apoptosis when VSMCs were co-incubated with CML and β-glycerophosphate (β-GP) for 48 h (Supplementary Figure 2). These results indicate that cell apoptosis is not the major cause of CML-induced VSMC calcification in the early stage.

### CML accelerates the progression of VSMC calcification

**Figure 2:**
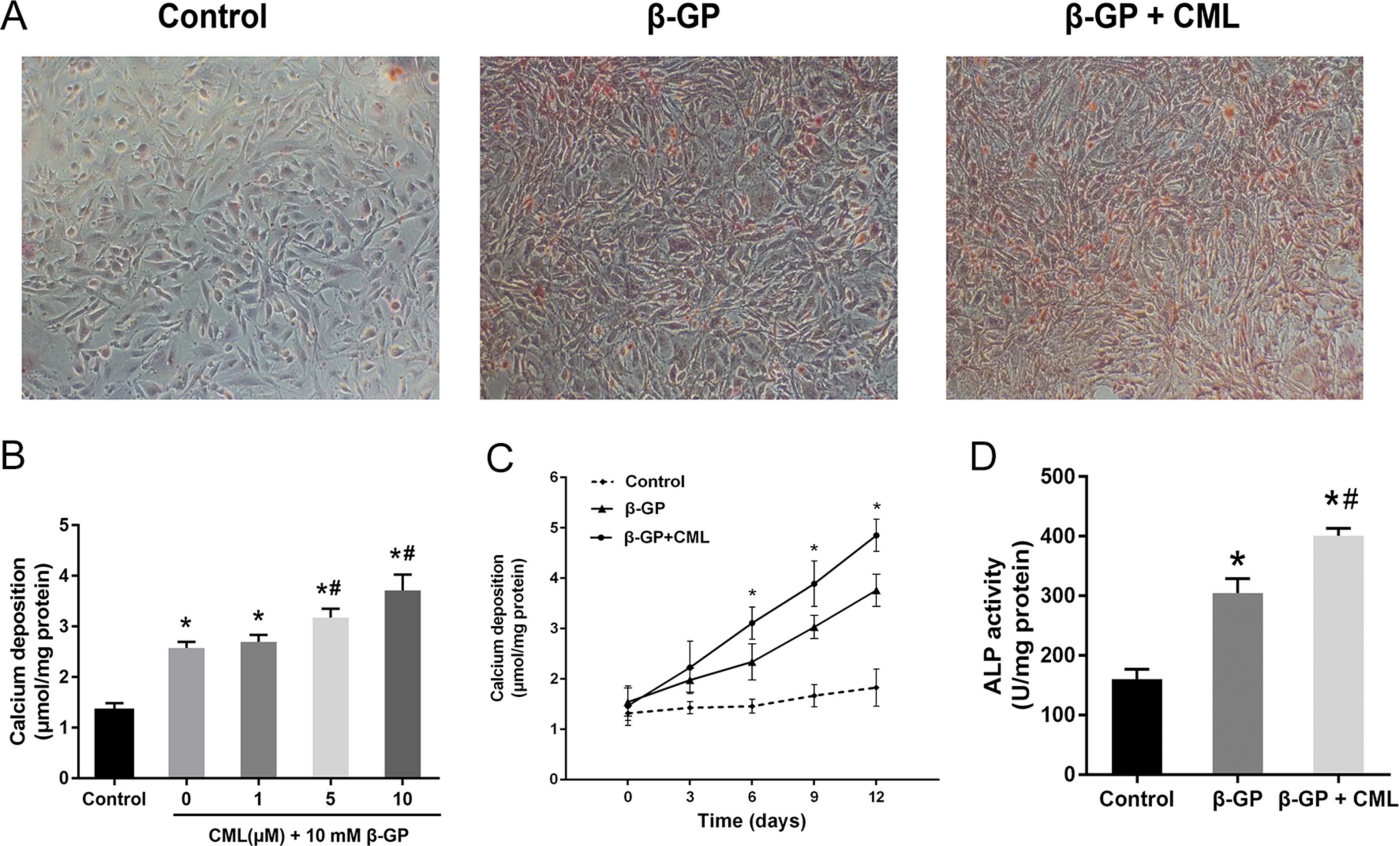
CML increased calcium deposition in VSMCs (**A**) VSMCs were cultured in the presence of 10 mM β-GP with or without the indicated concentrations of CML for two weeks with a medium change every two days. Calcium deposition was visualized by Alizarin Red S staining at the light microscopic level (100×). (**B**) VSMC calcification increased in a dose-dependent manner after incubation with 1, 5, and 10 μM CML for two weeks. Quantitative analysis of calcium deposition in VSMCs normalized to protein content. (**C**) VSMC calcification increased in a time-dependent manner after incubation with or without 10 μM CML for 0, 3, 6, 9, and 12 days. Quantitative analysis of calcium deposition in VSMCs normalized to protein content. (**D**) ALP activity was measured and normalized to protein content for quantitative analysis. ^*^P < 0.05 compared with the normal control group. ^#^*P* < 0.05 compared with the β-GP group.

Before investigating the role of PDK4 in the pathogenesis of diabetic vascular calcification, we first investigated the effects of CML on calcium deposition in VSMCs. VSMCs were cultured in the presence of 10 mM β-GP with or without the indicated concentrations of CML for two weeks. Microscopic observations revealed that calcium deposition in VSMCs was markedly increased by CML exposure, which was verified by morphological changes (nodule formation) (Figure 2A). Furthermore, CML stimulated calcium deposition in a dose- and time-dependent manner (Figure 2B and 2C). Additionally, ALP activity, an early indicator of the presence of calcium deposits, was enhanced by exposure to β-GP relative to the control group, and it increased further after incubation with CML (Figure 2D). Consistent with previous studies, these results suggest that CML accelerates VSMC calcification in a time- and dose-dependent manner.

### CML/RAGE signalling induced oxidative stress during VSMC calcification

**Figure 3:**
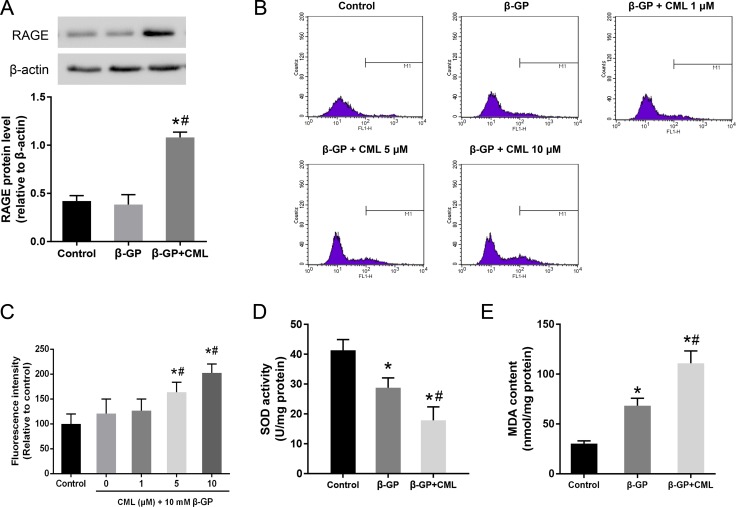
Activation of the CML/RAGE signalling axis promotes oxidative stress in VSMCs (**A**) VSMCs were treated with 10 mM β-GP with or without 10 μM CML for 24 h, and the expression of RAGE was analysed by western blot. (**B**, **C**) After VSMCs were treated with 10 μM CML for 4 h, ROS production was measured by flow cytometry. (**D**) SOD activity was measured. (**E**) MDA content was determined. ^*^P < 0.05 compared to the control group. ^#^*P* < 0.05 compared to the β-GP group.

It is known that the AGE-RAGE signalling pathway, which activates multiple intracellular signalling pathways, plays an important role in the pathogenesis of diabetic complications. We first detected the expression of RAGE in VSMCs. VSMCs were cultured in the presence of 10 mM β-GP with or without 10 μM CML for 24 h. As shown in Figure 3A, RAGE expression in the CML-treated group was significantly increased compared to that in the β-GP and control groups, which indicated that CML/RAGE signalling is involved in VSMC calcification. A significant association was not observed between the β-GP and control groups.

Since oxidative stress refers to elevated intracellular levels of ROS that damage cell components, flow cytometric measurements of ROS production were performed to estimate the degree of intracellular oxidative stress, and the results were expressed as the mean fluorescence intensity. We observed that ROS production was significantly increased in the β-GP + CML group compared to that in the control and β-GP groups (Figure 3B and 3C). Furthermore, CML induced ROS production in a dose-dependent manner. In addition, compared to the control group, the activity of superoxide dismutase (SOD) in the β-GP and β-GP + CML groups was significantly decreased (Figure 3D). The malondialdehyde (MDA) content was significantly increased in the CML + β-GP group compared to that in the β-GP and control groups (Figure 3E). Therefore, these data indicate that CML treatment increased ROS production and oxidative stress in a dose-dependent manner.

### PDK4 expression is enhanced during VSMC calcification

**Figure 4:**
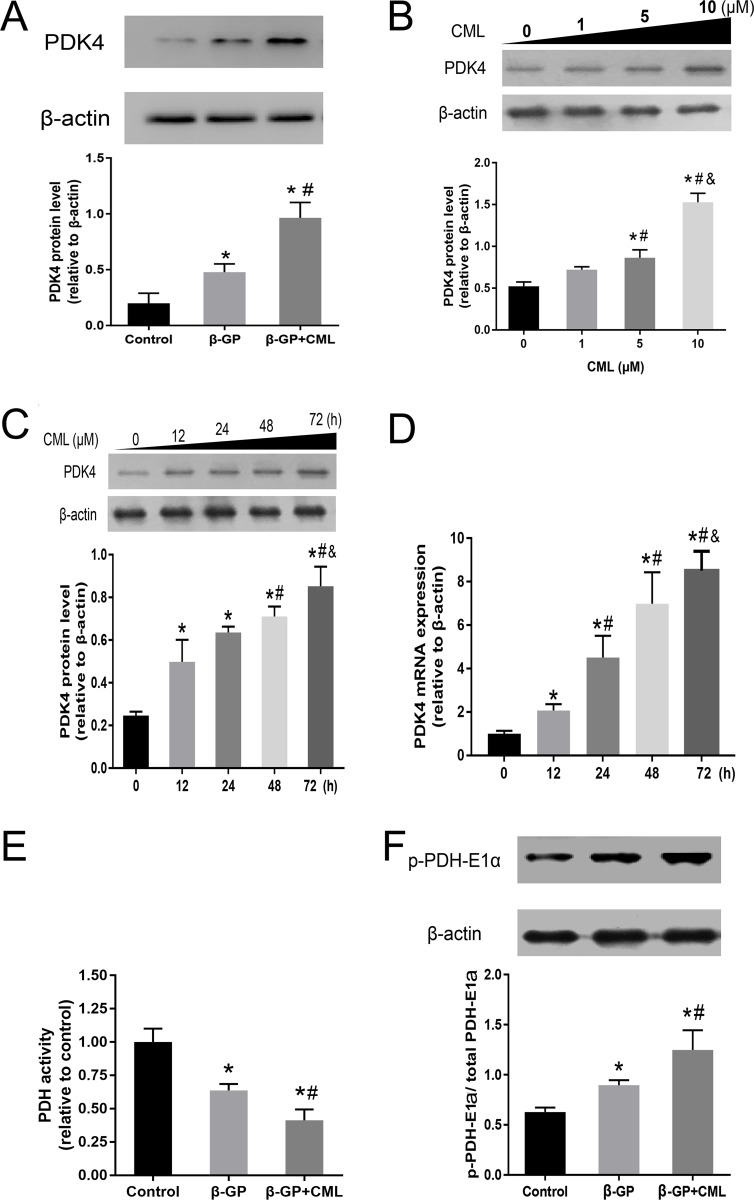
PDK4 expression is increased during VSMC calcification (**A**) VSMCs were cultured with calcium medium (β-GP) in the presence or absence of CML (10 μM) for 24 h, and PDK4 expression was analysed by western blot. ^*^P < 0.05 compared with the normal control group. ^#^*P* < 0.05 compared with the β-GP group. (**B**) VSMCs were treated with 0, 1, 5, and 10 μM CML for 24 h, and CML was shown to increase PDK4 expression in a dose-dependent manner. ^*^P < 0.05 compared with the normal control group. ^#^*P* < 0.05 compared with the CML (1 μM) group. ^&^ *P* < 0.05 compared with the CML (5 μM) group. (**C**) VSMCs were treated with 10 μM CML for 0, 12, 24, 48, and 72 h, and CML was shown to increase PDK4 expression in a time-dependent manner. ^*^P < 0.05 compared with the normal control group. ^#^*P* < 0.05 compared with the CML (12 h) group. ^&^ *P* < 0.05 compared with the CML (48 h) group. (**D**) PDK4 mRNA levels were determined using RT-qPCR. ^*^P < 0.05 compared with the normal control group. ^#^*P* < 0.05 compared with the CML (12 h) group. ^&^ *P* < 0.05 compared with the CML (48 h) group. (**E**) PDH activity was measured. ^*^P < 0.05 compared with the normal control group. ^#^*P* < 0.05 compared with the β-GP group. (**F**) VSMCs were cultured with calcium medium in the presence or absence of CML (10 μM) for 24 h, and the level of PDH-E1α phosphorylation was analysed by western blot. ^*^P < 0.05 compared with the normal control group. ^#^*P* < 0.05 compared with the β-GP group.

To detect whether PDK4 expression was related to CML-induced VSMC calcification, we first detected PDK4 expression by western blot. VSMCs were treated with 10 mM β-GP with or without 10 μM CML for 24 h, and we observed that the level of PDK4 protein was more significantly increased in the β-GP + CML group than in the β-GP and control groups, which indicated that PDK4 is involved in CML-induced VSMC calcification (Figure 4A).

To further investigate the effects of CML treatment on PDK4 protein expression, VSMCs were treated with 0, 1, 5, and 10 μM CML for 24 h, and the protein expression level of PDK4 in VSMCs was analysed by western blot. We observed that the protein expression level of PDK4 was increased in a dose-dependent manner (Figure 4B). Then, VSMCs were treated with 10 μM CML for 0, 12, 24, 48, and 72 h, and the protein and mRNA expression levels of PDK4 were subsequently analysed by western blot and quantitative real-time RT-PCR (RT-qPCR), respectively. The protein and mRNA expression levels of PDK4 were markedly increased with increasing incubation time, which indicated that CML effectively promoted PDK4 mRNA and protein expression in VSMCs in a time-dependent manner (Figure 4C and 4D). Together, these data suggest that CML increases the expression of PDK4 in a time- and dose-dependent manner.

Since PDKs control PDH activity via site-specific phosphorylation of the E1α subunits, we detected PDH activity using a microplate assay kit and the level of PDH-E1α (Ser300) phosphorylation in the presence of phosphatase inhibitors as indirect measurements of PDK activation. We observed that PDH activity was nearly 60% lower in the CML + β-GP group than in the control group (Figure 4E). Moreover, the level of phosphorylated PDH-E1α was increased in the CML + β-GP group compared to that in the control group, suggesting that CML treatment increases PDK4 expression (Figure 4F; Supplementary Figure 3). Thus, these data indicate that CML promotes the expression of PDK4 during VSMC calcification.

### PDK4 expression is correlated with the level of intracellular oxidative stress

**Figure 5:**
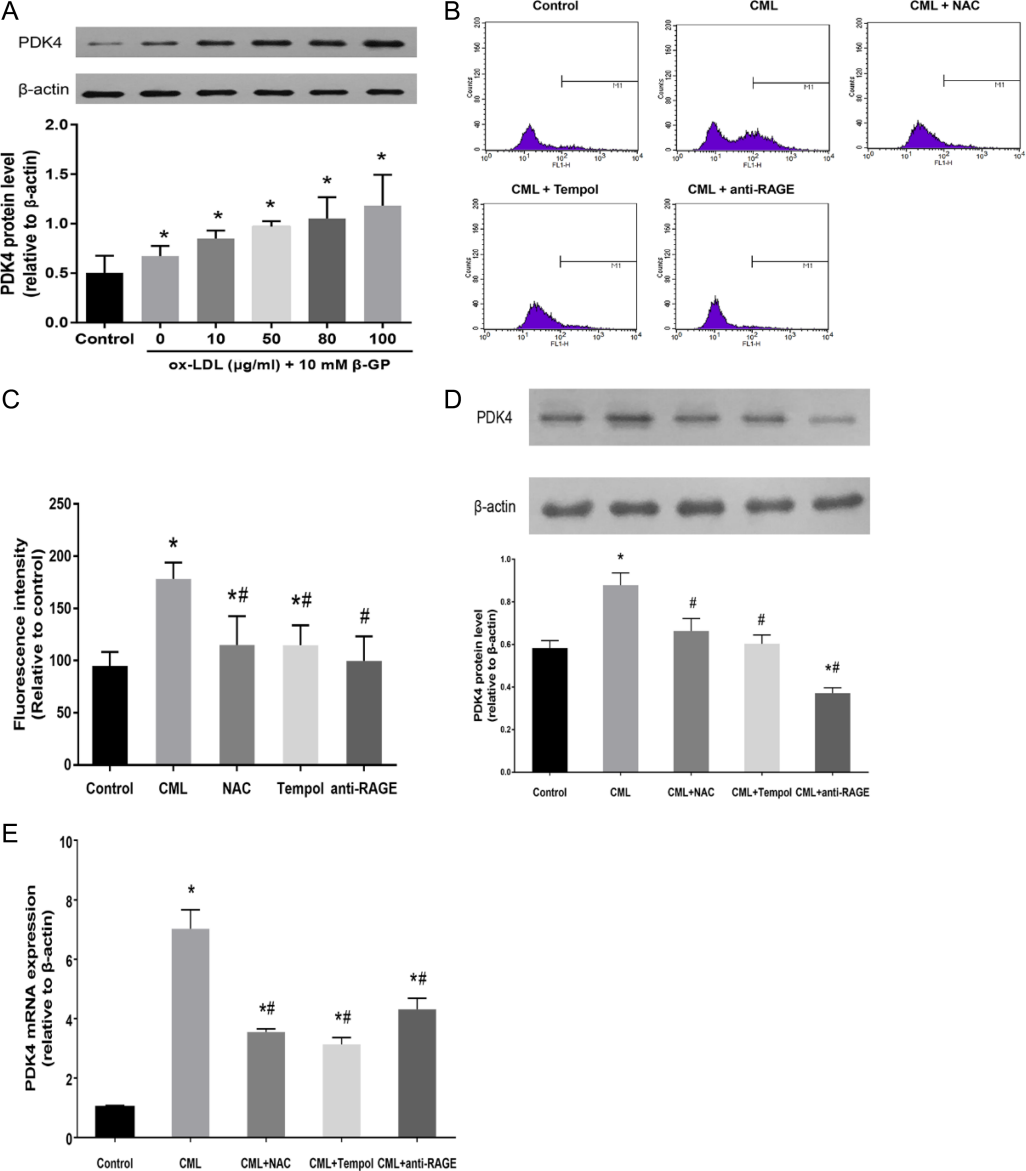
PDK4 expression is correlated with the level of intracellular oxidative stress (**A**) VSMCs were cultured with 10 mM β-GP and the indicated concentrations of ox-LDL for 4 h, and the expression of PDK4 was analysed by western blot. (**B**, **C**) VSMCs were preincubated with the indicated concentrations of NAC, tempol, and anti-RAGE antibody for 4 h, and the cells were then incubated with 10 μM CML. ROS production was measured by flow cytometry. (**D**, **E**) VSMCs were pretreated with antioxidants and anti-RAGE antibody and then exposed to CML for 24 h. PDK4 expression was analysed by western blot and RT-qPCR. ^*^P < 0.05 compared with the normal control group. ^#^*P* < 0.05 compared with the CML group.

As mentioned above, oxidative stress and ROS production were enhanced in calcified VSMCs following CML treatment in a dose-dependent manner. Oxidized low density lipoprotein (ox-LDL), which is enriched in atherosclerotic plaques, has been shown to generate oxidative stress in cardiovascular diseases [23, 24]. To better demonstrate that oxidative stress is linked to PDK4 expression, VSMCs were incubated with a wide range of ox-LDL concentrations (from 10 to 80 μg/ml) for 4 h as a positive control to induce oxidative stress conditions. We observed increased PDK4 expression following ox-LDL exposure in a dose-dependent manner (Figure 5A). These results further indicate that PDK4 expression is susceptible to the intracellular levels of *ROS.*

Next, we investigated whether inhibition of oxidative stress by antioxidants or an anti-RAGE antibody could suppress the expression of PDK4. VSMCs were pretreated with antioxidants, including N-acetylcysteine (NAC) and tempol, for 4 h followed by exposure to CML for 4 h. We observed that ROS generation in VSMCs, as demonstrated by flow cytometry, was significantly suppressed by co-incubation with either NAC or tempol (Figure 5B and 5C). In addition, the anti-RAGE antibody, which inhibits binding between RAGE and CML, was used to block the induction of the AGEs-RAGE axis and cellular oxidative stress in VSMCs by CML. We observed that the anti-RAGE antibody abolished the stimulatory effect of CML on PDK4 protein expression (Figure 5D), which indicates that inhibition of the CML/RAGE axis dramatically suppresses PDK4 protein expression. Supplementation with antioxidants, either NAC or tempol, also significantly reduced the expression of PDK4 (Figure 5D). Furthermore, the results of RT-qPCR further confirmed that oxidative stress is critically involved in the expression of PDK4 (Figure 5E).

### Effects of PDK4 inhibition and RAGE blockade on VSMC calcification

**Figure 6:**
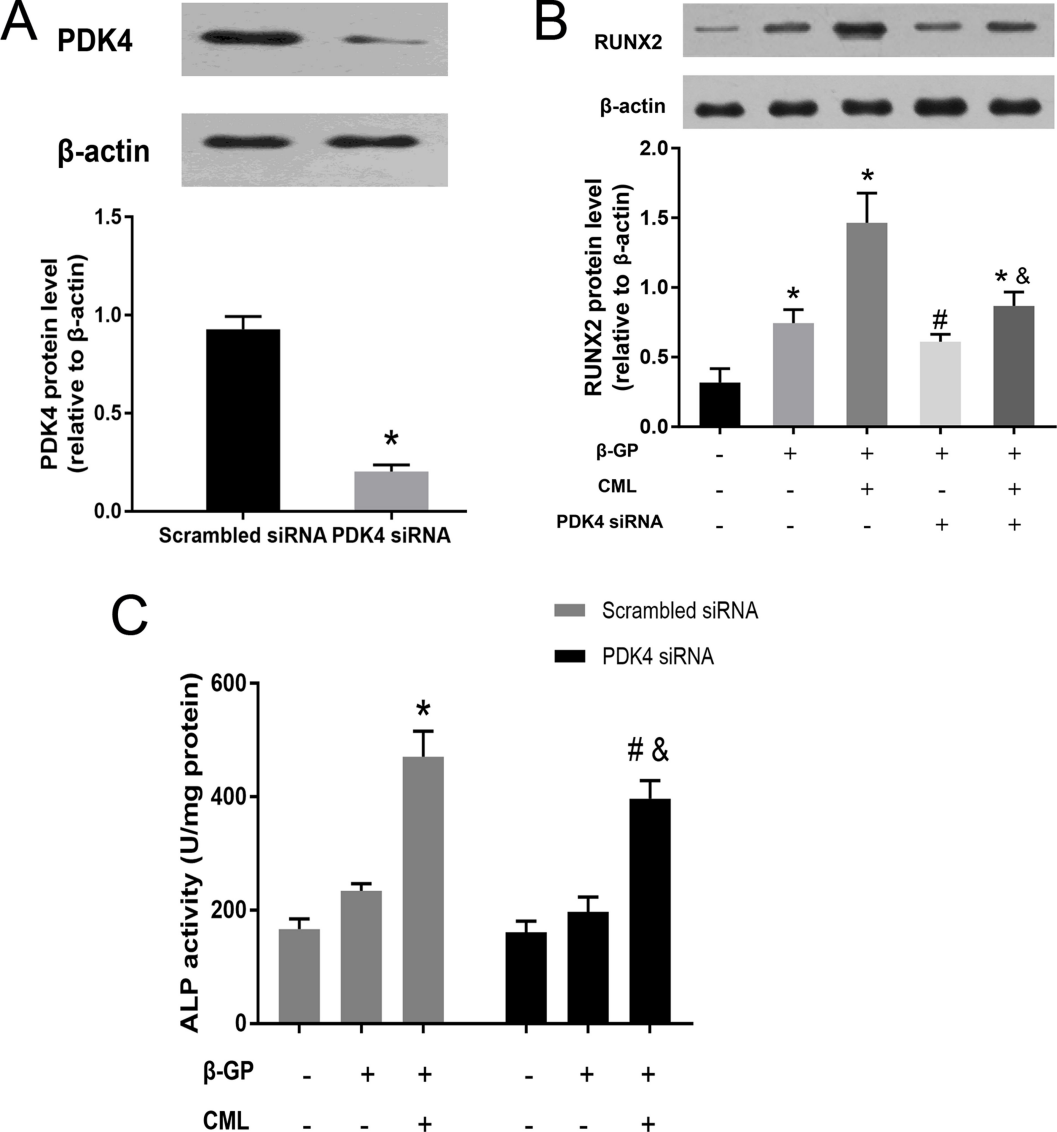
CML increased RUNX2 expression and ALP activity in VSMCs through a PDK4-dependent pathway (**A**) VSMCs were transfected with siRNA against PDK4 or scrambled siRNA for 48 h, and PDK4 expression was detected by western blot. ^*^P < 0.05 compared with the scrambled siRNA group. (**B**) VSMCs were treated with siRNA against PDK4 for 24 h and then cultured in the presence of 10 mM β-GP with or without 10 μM CML for 24 h. The effects of CML on RUXN2 expression were measured by western blot. ^*^P < 0.05 compared with the normal control group. ^#^*P* < 0.05 compared with the β-GP group. ^&^ *P* < 0.05 compared with the β-GP + CML group. (**C**) VSMCs transfected with siRNA against PDK4 or scrambled siRNA were treated with calcified medium plus CML, and then, ALP activity was detected. ^*^P < 0.05 compared with the control group (scrambled siRNA). ^#^*P* < 0.05 compared with the control group (PDK4 siRNA). ^&^ *P* < 0.05 compared with the β-GP + CML group (scrambled siRNA).

Since RUNX2 is an essential transcription factor for osteoblastic differentiation, we postulated that PDK4 may participate in RUNX2 upstream signalling in CML-induced VSMC calcification. To confirm this hypothesis, small interfering RNA (siRNA) targeting PDK4 was transfected into VSMCs. The knockdown efficiency was nearly 70% in VSMCs transfected with PDK4 siRNA compared to VSMCs transfected with scrambled *siRNA* (Figure 6A). While VSMCs incubated with 10 μM CML exhibited significantly increased RUNX2 expression, downregulation of PDK4 by siRNA potently prevented RUNX2 protein expression in VSMCs (Figure 6B). In addition, CML-induced ALP activity was significantly decreased by PDK4 siRNA (Figure 6C). These results suggest that PDK4 activation was responsible for RUNX2 expression and ALP activity in CML-induced VSMC calcification.

Then, to determine whether PDK4 inhibition or RAGE blockade can alleviate calcium deposition in VSMCs, VSMCs were co-incubated with the PDK inhibitor dichloroacetate (DCA) or anti-RAGE blocking antibody for two weeks. We observed that treatment with DCA or anti-RAGE blocking antibody inhibited the CML-induced increase in calcium deposition (Supplementary Figure 4). Taken together, our data suggest that CML/RAGE/PDK4 signalling plays an important role in diabetic vascular calcification.

### Effects of CML on metabolic changes during VSMC calcification

**Figure 7:**
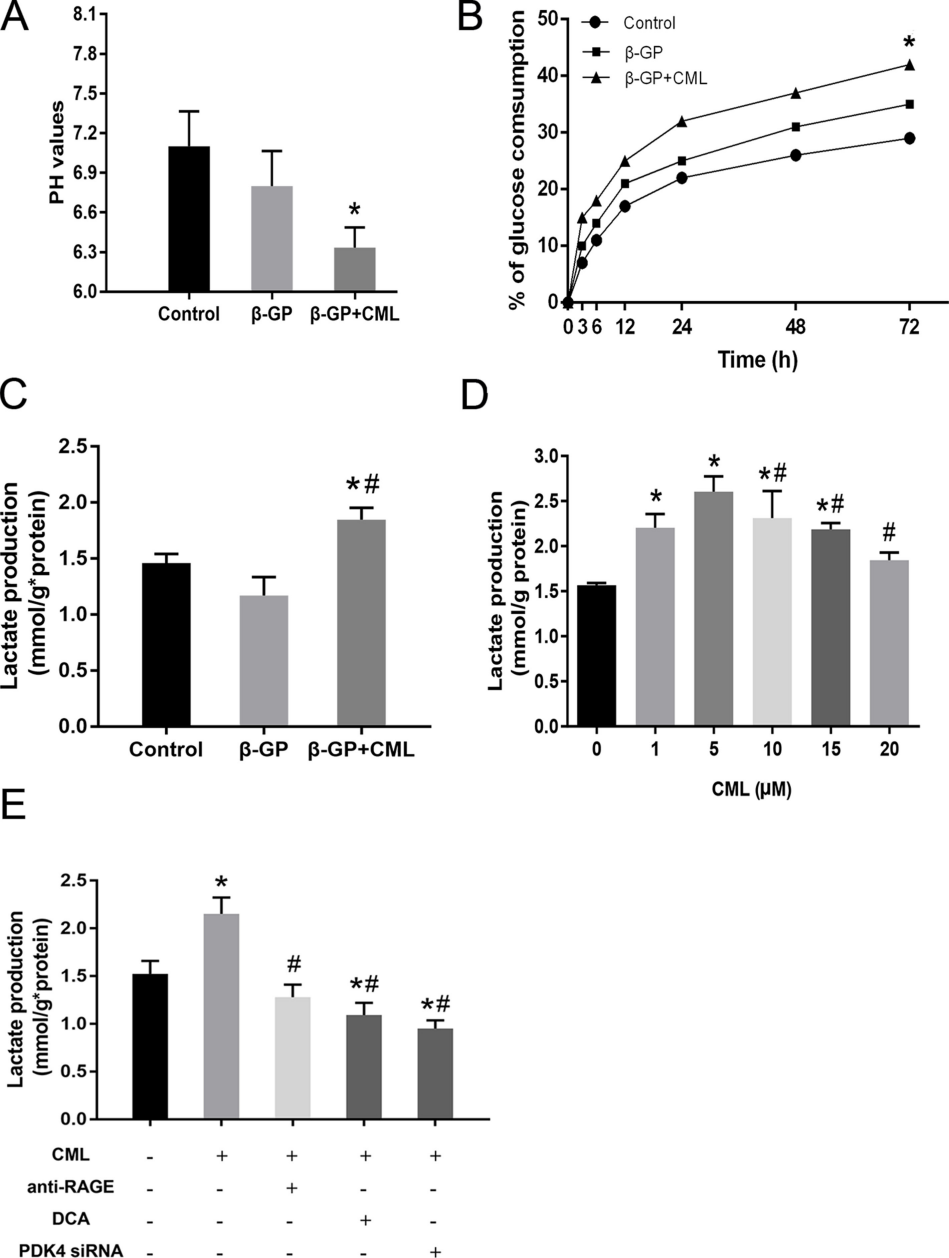
Effects of CML on metabolic changes during VSMC calcification (**A**) pH of the culture supernatant. After 48 h of growth, pH values were significantly lower in medium from cells treated with 10 μM CML. ^*^P < 0.05 compared with the normal control group. (**B**) Time-dependent changes in glucose consumption in VSMCs cultured in the absence or presence of 10 μM CML for different durations. ^*^P < 0.05 compared with the control group. (**C**) VSMCs were cultured in the presence of 10 mM β-GP with or without 10 μM CML for 72 h. The concentration of lactate was detected using a lactate assay kit and normalized to protein content. ^*^P < 0.05 compared with the control group. ^#^*P* < 0.05 compared with the β-GP group. (**D**) VSMCs were treated with 0, 1, 5, 10, 15, and 20 μM CML for 72 h. The concentration of lactate was detected and normalized to protein content. ^*^P < 0.05 compared with the control group. ^#^*P* < 0.05 compared with the CML (5 μM) group. (**E**) VSMCs were pretreated with DCA (PDK4 inhibitor), PDK4 siRNA, or an anti-RAGE antibody and then treated with 10 μM CML for another 72 h. The concentration of lactate was measured and normalized to protein content. ^*^P < 0.05 compared with the control group. ^#^*P* < 0.05 compared with the CML group.

The rate of glucose metabolism through the glycolytic pathway is tightly regulated and depends on the energetic and metabolic needs of VSMCs; glycolysis is also used to provide energy in osteoblasts [25]. Moreover, we noticed that growth medium supplemented with phenol red easily changed from pink to yellow during the induction of VSMC calcification, especially with calcium medium or co-incubation with CML, which is suggestive of lower pH levels and potential metabolic changes in these cells. Consequently, we attempted to explore the potential role of CML in glucose metabolism during VSMC calcification by assessing the change in pH, glucose consumption, and intracellular lactate concentration. After two days of growth, we observed that the medium was significantly more acidic in the CML + β-GP group than in the β-GP and control groups (Figure 7A; Supplementary Figure 5). A time-dependent increase in glucose consumption was also detected when VSMCs were maintained in the presence of 10 mM β-GP with or without 10 μM CML, and this increase was more pronounced after 72 h (Figure 7B). Although there was no significant difference in the intracellular lactate concentration between the β-GP and control groups after 72 h, addition of CML (10 μM) to the calcium medium increased lactate production compared to that in the β-GP and control groups, suggesting that the glycolysis rate was increased to meet the energy needs (Figure 7C). In addition, after VSMCs were incubated with 1, 5, 10, 15, and 20 μM CML for 72 h, we observed that low or normal CML concentrations more rapidly induced lactate production than high CML concentrations (15–20 μM) (Figure 7D). These results indicate that short-term CML treatment shifts glucose metabolism from oxidative phosphorylation to lactate production in VSMCs.

To further clarify whether the CML/RAGE-mediated induction of glycolysis is PDK4 dependent, we detected changes in lactate production to indirectly assess the pathway. As mentioned above, CML treatment increased glycolysis, and when we pretreated VSMCs with an anti-RAGE antibody, we observed that lactate production was inhibited 72 h after exposure to CML (Figure 7E; Supplementary Figure 6). Additionally, administration of DCA or PDK4 siRNA also inhibited CML-induced lactate production. These results suggest that PDK4 activation by CML/RAGE signalling alters glucose metabolism during VSMC calcification.

## DISCUSSION

**Figure 8:**
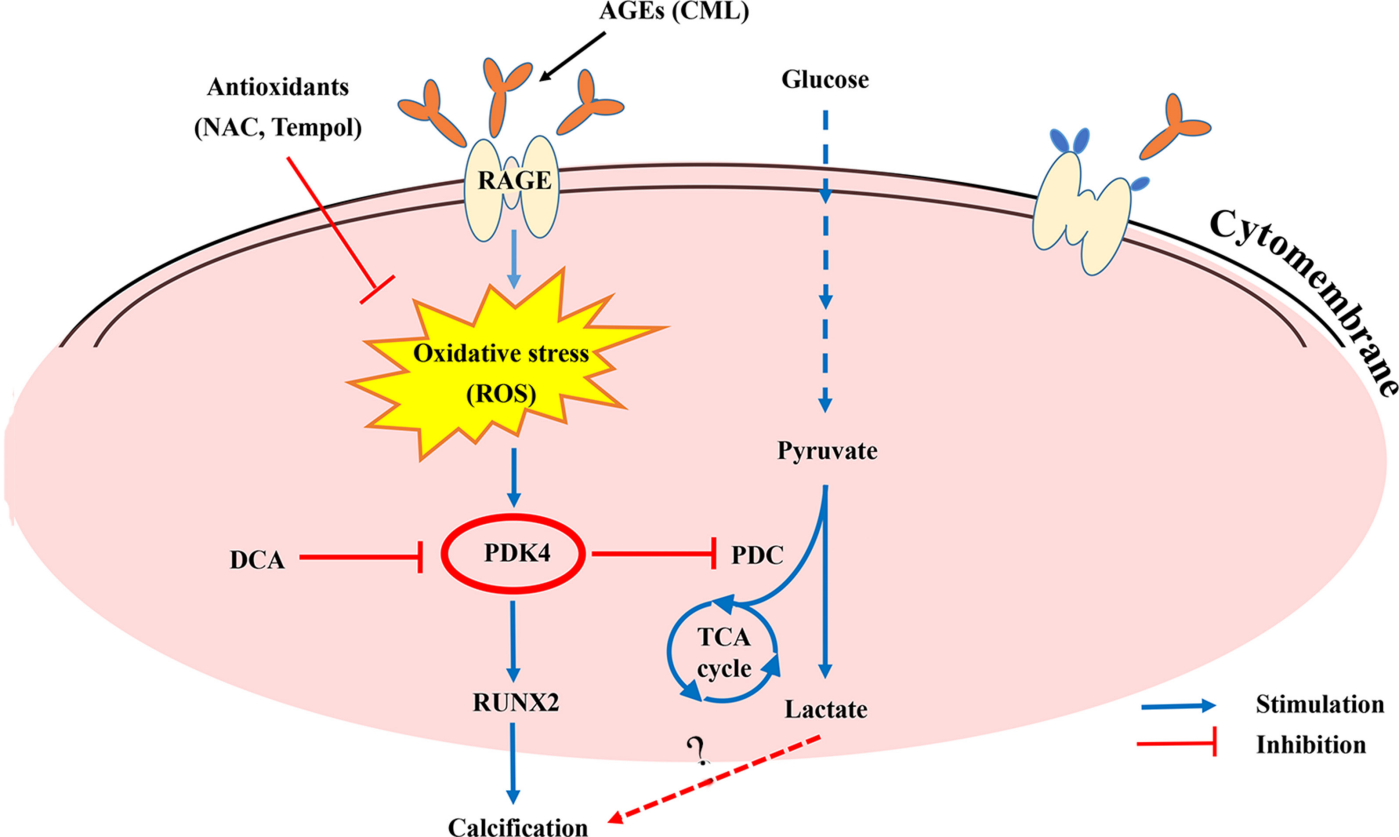
Summary of the working hypothesis of the study

Diabetes-mediated vascular calcification is strongly associated with an increased risk of cardiovascular events and death [7]. Excessive oxidative stress is commonly observed in the development of diabetic vascular complications, including both microvascular and cardiovascular complications [26]. Prior studies have implicated oxidative stress, AGEs, and metabolic shifts in the pathogenesis of vascular calcification [27, 28]. However, due to the complex nature of AGE/RAGE signalling and a cascade of complex downstream signals, the role of oxidative stress-mediated molecular signalling in the pathogenesis of diabetic vascular calcification has not been fully characterized. In our study, the results demonstrated that CML induced oxidative stress, which elevates the PDK4 expression and results in VSMC calcification via various signalling intermediates. Silencing of PDK4 by siRNA suppresses RUNX2 activation and ALP activity in CML-induced VSMCs. In addition, we identified a novel mechanism through which CML promotes glucose metabolism in VSMCs in a PDK4-dependent manner; in contrast, CML-induced glucose metabolism is hampered by PDK4 siRNA silencing or DCA treatment. Overall, our study suggests that PDK4 plays an integrative role in cellular metabolism and in stimulating calcium deposition in VSMCs (Figure 8).

ROS are signal molecules in tissue metabolism under conditions of oxidative stress, and physiological levels of ROS are essential for the governing of life processes through redox signalling; additionally, ROS serves as a signal molecule for the regulation of gene expression in different cell types [29, 30]. Since the mitochondrial respiratory chain is a major source of ROS production and PDK4 is an important mitochondria matrix enzyme that is dramatically changed by physiological conditions [18, 31], whether elevated intracellular ROS levels increase the expression of PDK4 is of interest. In our study, it was not unexpected to observe that either CML or ox-LDL promotes ROS production and enhances oxidative stress in calcified VSMCs, but we were the first to provide evidence that ROS functions as an upstream regulator of PDK4 expression and that this effect is alleviated by antioxidants or the anti-RAGE antibody. Our results also indicate that PDK4 is sensitive to local environmental factors. Two recent papers showed that PDK4 activation induces mitochondrial dysfunction, which leads to a further increase in ROS production [20, 32]. Therefore, there may be a reciprocal loop between ROS generation and PDK4 activation.

RUNX2, which is commonly present in calcified vasculature, is considered an essential transcription factor for osteogenic gene expression [33, 34]. Since PDK4 expression is increased in calcified vessels of patients with atherosclerosis, we explored whether PDK4 promotes the expression of several osteogenic genes. In this study, we showed that RUNX2 expression is strongly increased by PDK4 activation and that the CML/RAGE/oxidative stress axis is important for the pathogenesis of diabetic vascular calcification. Inhibition of PDK4 by siRNA significantly reduced RUNX2 expression and attenuated ALP activity. Our findings are consistent with those of Lee *et al.* [20], which suggested that PDK4 is critical for osteoblastic transdifferentiation during VSMC calcification. In contrast, a previous study reported that PDK4 positively regulates bone loss by promoting osteoclastogenesis in osteoblastic cells [35]. Different cell types and stimuli may explain the discrepancy between the studies.

Glucose metabolism is the predominant route for acquiring cellular energy, especially for VSMCs. Enhanced glucose metabolism is present during atherogenesis [36]. Several reports have shown that impairment of glucose metabolism is implicated in the pathogenesis of cardiovascular diseases [37]. In addition, Idelevich *et al.* [38] reported a high rate of glucose breakdown in cartilage and calcified vasculature. In our research, we observed that CML supplementation increases glucose uptake and glycolytic rates in VSMCs. The CML/RAGE-mediated induction of glycolysis is PDK4 dependent, which may be a novel therapeutic target for the treatment of vascular calcification. These findings are consistent with those of previous studies that show knocking out PDK4 results in the attenuation of glycolysis [39]. Abnormally high lactate levels, which indicate oxidative capacity, were observed in diabetic patients [40]. Limited available data indicate that blood lactate is likely associated with carotid atherosclerosis independent of other cardiovascular risk factors [41]. Recently, Wu and colleagues reported that lactate induces osteoblast differentiation in a pre-osteoblast cell line [42]. Generally, lactate accumulation represents energy inefficiency or metabolic impairment, which is partially due to insufficient oxygen delivery [43], as lactate accumulation is a measure of the gap between energy expenditure and oxidative capacity. We noted that CML promotes lactate production in calcified VSMCs and that this phenomenon is more effective at low doses of CML. Similar results have not been reported in the literature. Possible explanations might be that a high dose of CML mediates mitochondrial energy metabolic dysfunction, thus partly limiting glycolysis in VSMCs. Recently, Li *et al*. [43] reported that AGEs can disrupt mitochondrial energy metabolism and glycolysis. Further investigation of this fundamental mechanism of metabolic regulation will allow us to better understand diabetic vascular calcification.

In summary, we have demonstrated the pivotal role of PDK4 in CML-induced VSMC calcification, which occurs through the promotion of osteoblastic transdifferentiation and an increase in ALP activity as well as the alteration of glucose metabolism in VSMCs. ROS serves as an upstream mediator that induces PDK4 expression. Downregulation of PDK4 expression alleviates VSMC calcification. In addition, glucose consumption is increased in calcified VSMCs, and PDK4 activation is mediated by CML/RAGE signalling; moreover, PDK4 accelerates lactate production, which is blunted by DCA or PDK4 siRNA, indicating that there is a glucose metabolic switch in calcified VSMCs. Thus, targeting PDK4 or PDK4-regulating signals in VSMCs may be beneficial for the prevention of diabetic vascular calcification.

## MATERIALS AND METHODS

### Ethics statement

All animal studies were approved by the Ethics Committee of Southeast University. The studies were conducted in accordance with the guidelines for the care and use of laboratory animals published by the China National Institutes of Health.

### Reagents and antibodies

NAC was purchased from Biotechnology (Jiangsu, China). Ox-LDL was acquired from Yiyuan Biotechnology (Guangzhou, China). Anti-RUNX2 and anti-phospho PDHE1-A type I (Ser300) antibodies were obtained from Sigma–Aldrich (St. Louis, MO, USA). Anti-RAGE antibody was obtained from R&D systems (Minneapolis, MN, USA). Antibodies against PDK4 and β-actin were purchased from Abcam (Cambridge, MA, USA). The Quantichrom calcium assay kit was purchased from Biosino Bio-Technology and Science (Beijing, China). The ALP assay kit, bicinchoninic acid (BCA) protein assay kit, MTT assay kit, and ROS assay kit were purchased from Beyotime Biotechnology (Jiangsu, China). β-GP, tempol, and DCA were obtained from Sigma–Aldrich (St. Louis, MO, USA). The PDH enzyme activity microplate assay was purchased from Abcam (Cambridge, MA, USA). CML was purchased from Toronto Research Chemicals Inc. (Toronto, Canada). The glucose assay kit and lactate assay kit were purchased from the Jiancheng Bioengineering Institute (Nanjing, China).

### Cell culture

Primary VSMCs were isolated from the thoracic aortas of 5-to 6-week-old male Sprague Dawley rats according to previously described protocols [44]. VSMCs were cultured in Dulbecco's Modified Eagle's Medium (DMEM) supplemented with 20% foetal bovine serum and antibiotics at 37°C in a humidified atmosphere with 5% CO_2_. Cells between passages 3 and 8 were used in this study.

### Detection of intracellular ROS

Intracellular ROS levels were measured using an oxidation-sensitive fluorescent probe, 2,7-dichlorofluorescein diacetate (DCFH-DA). The treated cells were washed twice with serum-free DMEM and then incubated in the dark with 10 μM DCFH-DA for an additional 30 min. Images of fluorescent DCFH-DA were obtained using fluorescence microscopy.

### Calcification staining and measuring

Alizarin Red S staining was performed to show the calcification of VSMCs. VSMCs were washed twice with cold phosphate-buffered saline (PBS), fixed with 95% ethanol for 20 min, and then stained with 1% Alizarin Red S (pH 7.0) for 20 min at room temperature. Then, excess dye was removed by washing the cells with distilled water. Calcium deposition in VSMCs was measured using the Quantichrom calcium assay kit and normalized to the protein content determined using the BCA protein assay kit.

### ALP activity assay

ALP activity in VSMCs was measured using an ALP assay kit according to the manufacturer's instructions. The results were normalized to the total protein concentration.

### Measurement of VSMC viability

Cell viability was measured using a modified MTT assay. Briefly, VSMCs were seeded at a density of 5,000 cells/well in flat-bottomed 96-well culture plates. Then, VSMCs were treated with 0, 1, 5, 10, and 20 μM CML for 24, 48, and 72 h. After removing the medium, 10 μL of MTT was added to each well and incubated for 4 h at 37°C. The culture medium was then removed, and MTT formazan crystals were dissolved in 100 μL of dimethyl sulfoxide (DMSO). Absorbance was measured at a wavelength of 570 nm using a microplate reader.

### Transient transfection of siRNA

The PDK4 gene was silenced by a specific targeting siRNA duplex consisting of oligos with the sequences 5'-CCGUCUCUACGCCAAGUAUTT-3' and 5'-AUACUUGGCGUAGAGACGGTT-3'. Scrambled si RNA duplex oligos with the sequences 5′-UUCUC CGAACGUGUCACGUTT-3′ and 3′-TTAAGAGGCU UGCACAGUGCA-5′ were used as a negative control and did not target any genes. VSMCs were seeded onto 6-well plates, cultured for 24 h, and then transfected with siRNA duplexes against human PDK4 or negative control siRNA using ribo FECT^™^ CP transfection reagents according to the manufacturer's instructions.

### Measurement of glucose consumption and lactate production

To measure the consumption of glucose, supernatant was collected from cultured VSMCs at different time points. A glucose assay kit was used to detect the glucose concentration in culture supernatants according to the manufacturer's instructions; glucose levels were quantified by measuring absorption at 450 nm. Lactate production was measured using a lactate assay kit according to the manufacturer's instructions, and the relative values were normalized to protein content.

### RT-qPCR

RT-qPCR was performed to detect the mRNA expression levels of PDK4 and RUNX2. Total RNA was extracted from VSMCs using TRIzol reagent according to the manufacturer's instructions. Then, 1 μg of RNA was reverse transcribed using the Omniscript Reverse Transcription kit according to the manufacturer's protocol. RT-qPCR was performed using a ViiA7 Real-Time PCR system (Applied Biosystems, Foster City, CA, USA) and the QuantiNova SYBR-Green PCR kit according to the manufacturer's instructions. The primers for the target genes investigated in the present study are shown in Table 1. The standard PCR conditions consisted of 95°C for 5 min and 40 cycles of 95°C for 30 s, 58°C for 30 s, and 72°C for 45 s. The expression levels of target genes were determined and normalized to the expression level of β-actin. All samples were amplified in triplicate. Relative mRNA expression levels of target genes were calculated using the 2-ΔΔCt method.

**Table 1:** Primer sequences used in the RT-qPCR analysis

Genes	Primer sequences
PDK4	Forward, 5'- CAGACAGAGGAGGTGGTGTT-3'
	Reverse, 5'-CGAGAAATTGGCAAGCCGTA-3'
RUNX2	Forward, 5'-CAGAAGGCACAGACAGAAGC-3'
	Reverse, 5'- AGGACTTGGTGCAGAGTTCA -3'
β-actin	Forward, 5′-GGCTGTATTCCCCTCCATCG-3′
	Reverse, 5′-CCAGTTGGTAACAATGCCATGT-3′

### Western blot analysis

VSMCs were lysed in RIPA buffer. Protein concentration was measured using a BCA protein assay kit. Then, 20 μg of total protein was loaded onto a 12% SDS-PAGE gel and transferred onto nitrocellulose membranes. After blocking the membranes with 5% non-fat milk for one hour, the membranes were incubated with primary antibodies overnight at 4°C and subsequently incubated with horseradish peroxidase (HRP)-labelled secondary antibodies (1: 2,000 dilution) for 1 h at room temperature. HRP-conjugated secondary antibodies were applied in conjunction with an ECL chemiluminescence detection system. Protein expression was analysed by Gel-Pro Analyzer 4 software and normalized to β-actin.

### Statistical analysis

All data are presented as the mean ± standard deviation (SD) of three independent experiments. Data were analysed and plotted using GraphPad Prism software (GraphPad Prism 5.0; GraphPad Software Inc., La Jolla, CA, USA). One-way ANOVA followed by the Bonferroni test was used to compare multiple groups, and Student's *t*-test was used to compare the means of two groups. A *P*-value less than 0.05 was considered to be statistically significant.

## SUPPLEMENTARY MATERIALS FIGURES


