# Supplementary material for: Nε-carboxymethyl-lysine promotes calcium deposition in VSMCs via intracellular oxidative stress-induced PDK4 activation and alters glucose metabolism
Source: Oncotarget. 2017 Dec 1;8(68):112841–54. doi: 10.18632/oncotarget.22835 (PMC5762555; doi:10.18632/oncotarget.22835)
Supplement: Supplementary file 1 [file oncotarget-08-112841-s001.pdf]

Nε-carboxymethyl-lysine promotes calcium deposition in VSMCs via intracellular oxidative stress-induced PDK4 activation and alters glucose metabolism

SUPPLEMENTARY MATERIALS

Detection of cell apoptosis by flow cytometry

The rate of apoptosis was determined by measuring surface expression of phosphatidylserine with an Annexin V-FITC/propidium iodide (PI) apoptosis detection kit according to the manufacturer's instructions. After VSMCs were seeded onto 6-well plate for 24 h, VSMCs were treated with 10 μM CML for 24, 48, and 72 h. Then, the cells were collected and washed with ice-cold phosphate-buffered saline (PBS) three times. A total of 5×10^5 cells was resuspended with 500 μL of binding buffer containing Annexin V and PI for 20 min at 37°C in the dark. The number of viable, apoptotic and necrotic cells were quantified by a flow cytometer. The apoptosis rate (%) = (the number of apoptotic cells / the number of total cells) × 100%.

TUNEL assay

Apoptosis was detected using the TUNEL assay. Briefly, VSMCs cultured on coverslips in 6-well plates were fixed in 4% paraformaldehyde. Then, VSMCs were pre-digested with proteinase K for 15 min at room temperature and incubated in PBS containing 3% H_2O_2 for 10 min to block endogenous peroxidase activity. Then, VSMCs were incubated in equilibration buffer and treated with terminal deoxynucleotidyl transferase (TdT) enzyme for 60 min at room temperature. After washing, cells were incubated with streptavidin horseradish peroxidase (HRP) in the presence of TdT for 1 h at 37°C and stained by 3,3′-diaminobenzidine (DAB). After washing twice with PBS, the number of TUNEL-positive cells and total cells were counted under a light microscope.

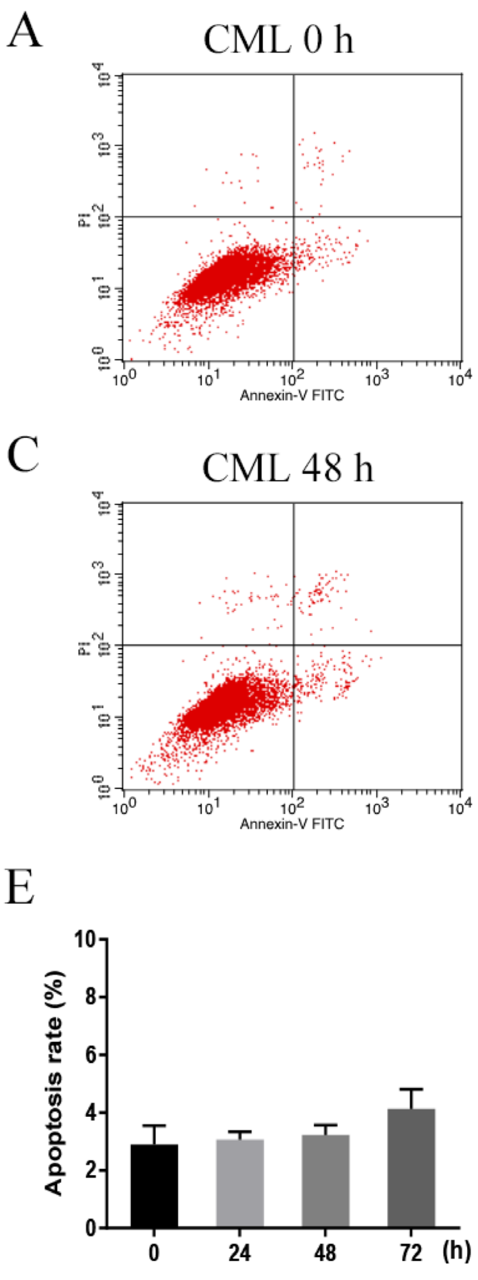

Supplementary Figure 1: Flow cytometry analysis of apoptosis in CML-induced VSMCs. VSMCs were treated with 10 μM CML for 0, 24, 48, and 72 h. (**A**–**D**) Representative data demonstrating Annexin V/PI double staining of VSMCs treated with 10 μM of CML for 0, 24, 48, and 72 h. (**E**) Data analysis of A-D.

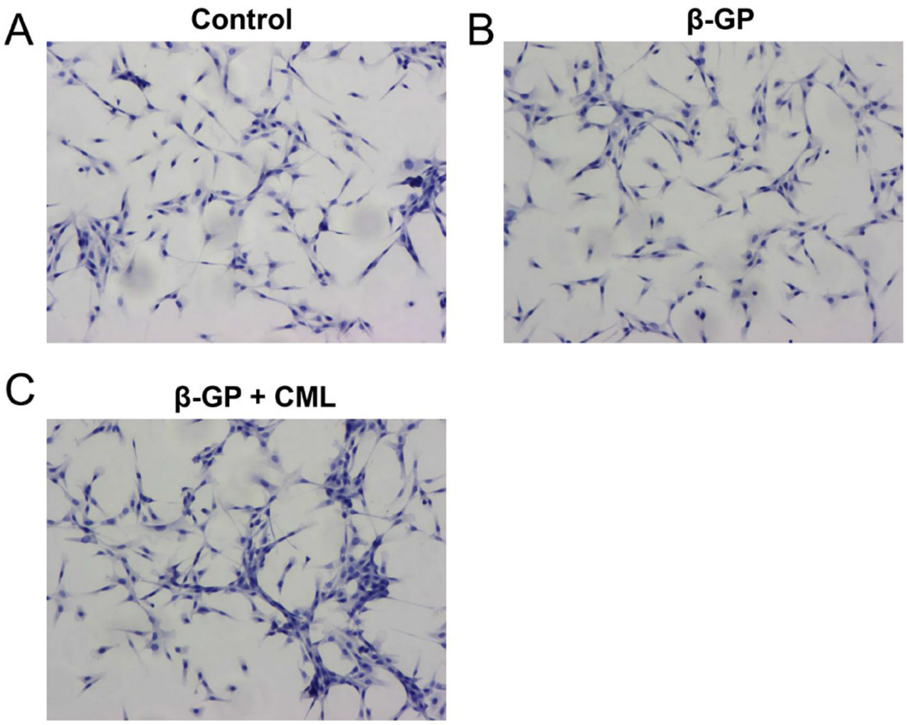

Supplementary Figure 2: The percentage of apoptotic cells as assessed by TUNEL staining (100×). (**A**) Control. (**B**) β-GP. (**C**) β-GP + CML.

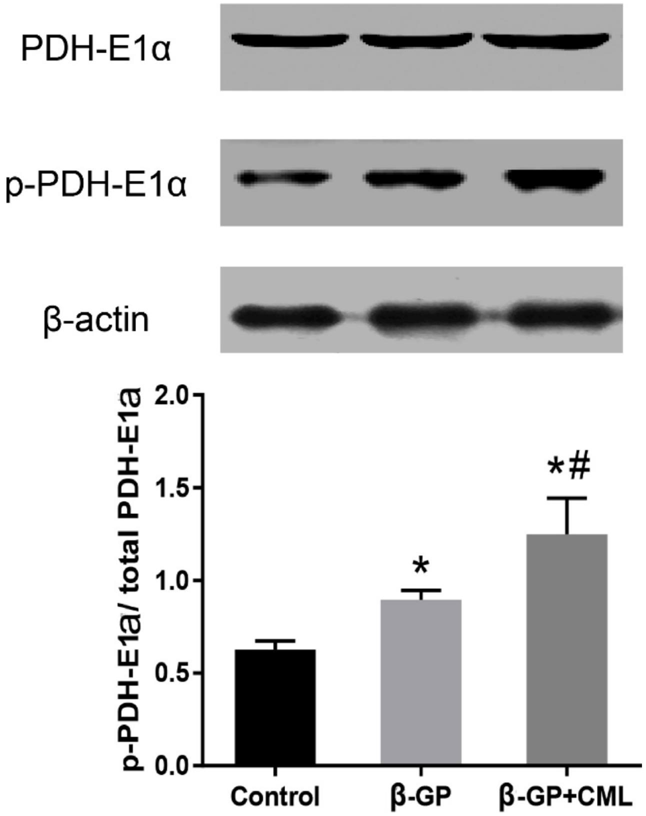

Supplementary Figure 3: The effect of CML on PDH-E1α expression in VSMCs. VSMCs were cultured in the presence of 10 mM β-GP with or without 10 μM CML for 24 h, and the protein expression level of PDH-E1α was analysed by western blot.

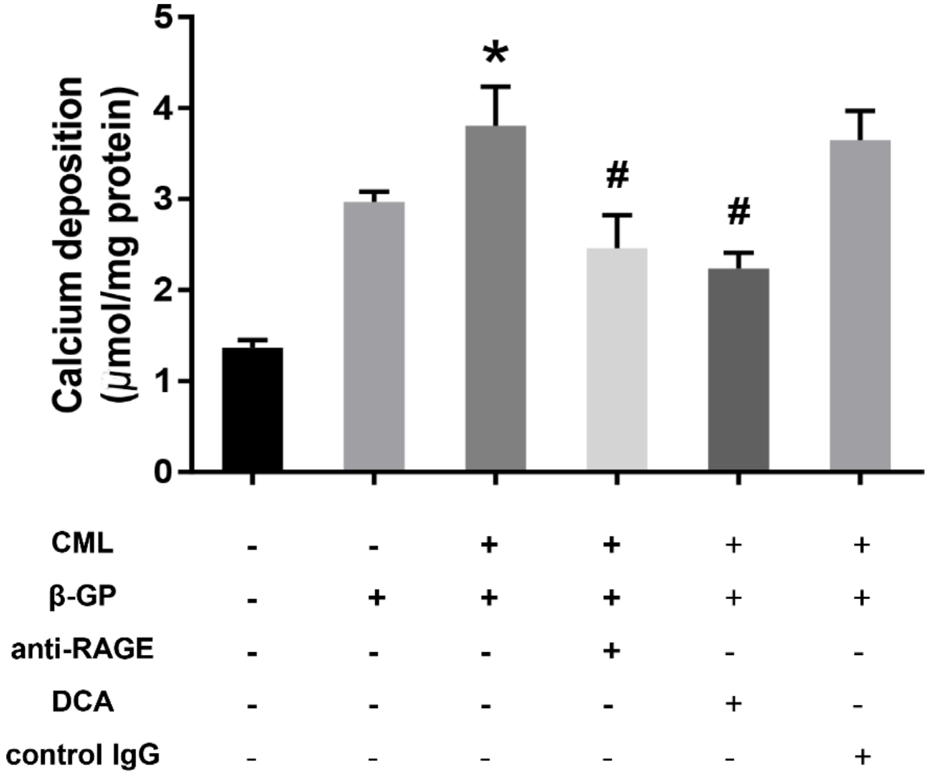

Supplementary Figure 4: Effects of DCA treatment and RAGE blockade on VSMC calcification. Cells were cultured in the presence of 10 mM β-GP and 10 μM CML with the indicated concentrations of DCA or anti-RAGE for two weeks. $^{*}P < 0.05$ compared with the control group. $^{\#}P < 0.05$ compared with the CML + β-GP group.

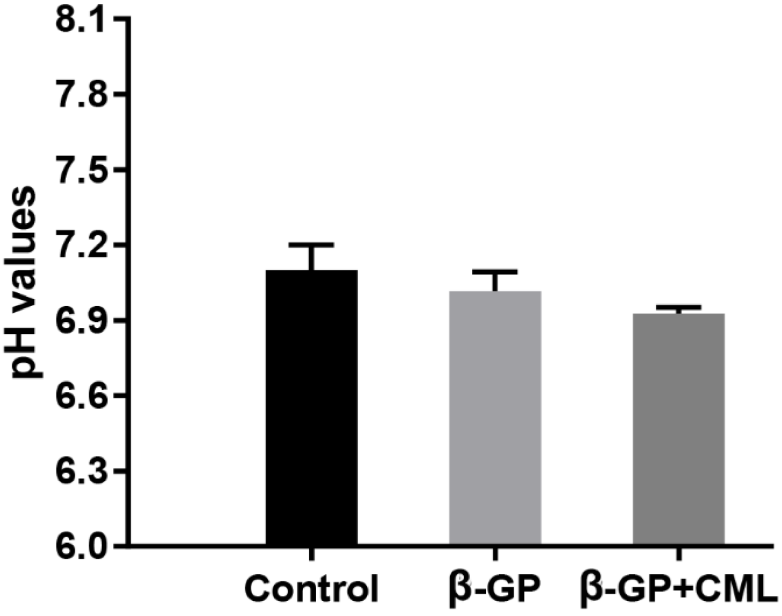

Supplementary Figure 5: The pH values of the culture supernatants were determined on day 1. No significant changes in pH values were observed among the three groups.

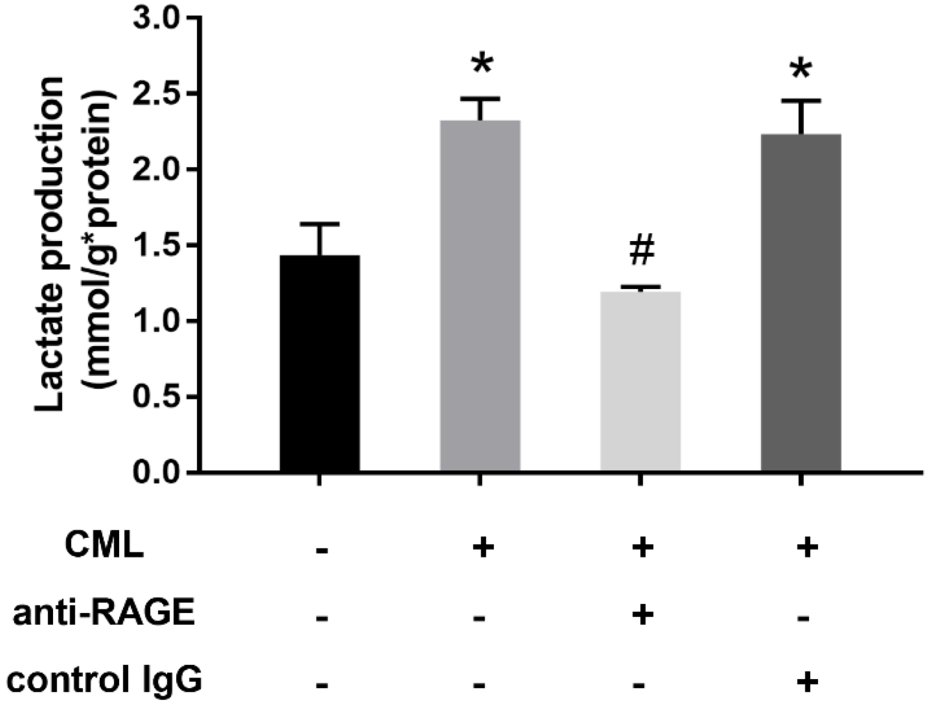

Supplementary Figure 6: Effects of RAGE blockade on lactate production. VSMCs were pretreated with anti-RAGE antibody or non-specific IgG and then treated with 10 μM CML for another 72 h. The concentration of lactate was measured and normalized to protein content. $^{*}P < 0.05$ compared with the control group. $^{\#}P < 0.05$ compared with the CML group.
